# Dangerous Behavior and Intractable Axial Skeletal Pain in Performance Horses: A Possible Role for Ganglioneuritis (14 Cases; 2014–2019)

**DOI:** 10.3389/fvets.2021.734218

**Published:** 2021-12-10

**Authors:** Melinda R. Story, Yvette S. Nout-Lomas, Tawfik A. Aboellail, Kurt T. Selberg, Myra F. Barrett, C. Wayne Mcllwraith, Kevin K. Haussler

**Affiliations:** ^1^Department of Clinical Sciences, Orthopaedic Research Center, College of Veterinary Medicine and Biomedical Sciences, Colorado State University, Fort Collins, CO, United States; ^2^Department of Microbiology, Immunology, and Pathology, College of Veterinary Medicine and Biomedical Science, Colorado State University, Fort Collins, CO, United States; ^3^Department of Environmental and Radiological Health Sciences, College of Veterinary Medicine and Biomedical Science, Colorado State University, Fort Collins, CO, United States

**Keywords:** horse, poor performance, neuropathic pain, epidural hemorrhage, ganglionitis

## Abstract

**Introduction:** Dangerous behavior is considered an undesired trait, often attributed to poor training or bad-tempered horses. Unfortunately, horses with progressive signs of dangerous behavior are often euthanized due to concerns for rider safety and limitations in performance. However, this dangerous behavior may actually originate from chronic axial skeleton pain. This case series describes the medical histories and clinical presentations of horses presented for performance limitations and dangerous behavior judged to be related to intractable axial skeleton pain.

**Material and Methods:** Fourteen horses that developed severe performance limitations resulting in euthanasia were included. A complete spinal examination and behavioral responses, gait and neurologic evaluations, diagnostic imaging, gross pathologic and histopathologic examinations of the axial skeleton were performed on all horses. A tentative diagnosis of the affected spinal region was formulated using medical records, owner and trainer complaints, and antemortem examination findings. The selected spinal regions were further examined with gross and histopathologic evaluations of the associated osseous, soft tissue and neural tissues.

**Results:** Ten horses showed severe behavioral responses during the myofascial and mobilization examinations. Based on an aggregate evaluation, the cervicothoracic and lumbosacral regions were the most common regions believed to be the primary area of concern. All horses had moderate to severe ganglionitis present at multiple vertebral levels. Subdural and epidural hemorrhage or hematomas were a common finding (71%) in the cervicothoracic and lumbosacral regions.

**Discussion:** In this case series, neuropathic (i.e., structural) pain was judged to be the underlying cause of dangerous behavior. The dorsal root ganglia (DRG) serve an important role in relaying peripheral sensory information to the central nervous system and ganglionitis has been associated with neuropathic pain syndromes. This series highlights the need for more in-depth understanding of pain behavior and its clinical presentation and progression in chronic or severely affected horses. Limitations of the study are the lack of age-matched control DRG and the incomplete collection of DRG from every vertebral level of interest.

## Introduction

It is all too common that a rider purchases a new horse with excitement and high aspirations, but because of health or training issues, those expectations are never realized. These training limitations sometimes progress to dangerous behavior such as kicking out, refusing to go forward, bucking and rearing. The horse is then characterized as a problem horse, and training methods may become more punitive. Undesired behavior in horses most often stems from their attempt to avoid fear or pain (1, 2). Horses may develop undesirable traits that progress to dangerous behavior for a multitude of reasons that include lack of clear communications or use of aids, improper training, lameness (3), poor saddle fit (4), axial skeletal pain (5), and gastrointestinal or reproductive abnormalities (6). Some trainers acknowledge that the bad behavior may stem from undiscovered physical problems and they enlist the help of medical professionals. Routine therapies are often applied, and the horse is asked to go back to work; however, the behavioral concerns continue or are only alleviated for short periods of time. A therapeutic trial of non-steroidal anti-inflammatory (NSAIDs) medication over several days followed by ridden exercise can be used to determine whether bad behavior may have an underlying inflammatory or pain component (7). Although a negative response (i.e., no improvement to NSAIDS) does not preclude the presence of pain (8), this response may reinforce the perception that the affected horse has behavioral issues and needs more aggressive training. Unfortunately, this approach may exacerbate the underlying pain behavior. The horse may become more dangerous until the owner or trainer eventually give up and sell the horse; only for the process to be repeated with a new trainer and veterinarian. After much expense, and long durations of frustrating diagnostics and trials of ineffective treatments, owners may finally opt to euthanize the horse; for the safety of the rider and the well-being of the horse. If these horses are euthanized, a routine necropsy (9) often fails to provide any additional insights as to the cause of the dangerous behavior. The owners are eventually left wondering whether euthanasia of their horse was justified due to the lack of clinically significant pathologic findings.

Over a period of 5 years, the authors have identified a group of young to middle aged performance horses that became difficult to train and ultimately dangerous to ride within a short time after purchase. These horses were often very well-behaved and easy to handle for general care. However, when asked to work under tack or advance in training, they showed dangerous behavior such as bucking, refusing to go forward, and rearing to the extent that they were too dangerous to ride. After repeated and extensive musculoskeletal evaluations which included diagnostic anesthesia, diagnostic imaging, and the lack of response to numerous applied therapies, these horses were euthanized due to their dangerous behavior. This paper reports a series of 14 affected sports horses (representing 0.001% of horses presenting for evaluation in the same time frame), including performance history and previous lameness evaluations (retrospective component); and a detailed behavioral, physical, lameness and neurologic examination, diagnostic imaging, and gross and histologic examination of the vertebrae, spinal cord and dorsal root ganglia (prospective portion).

## Materials and Methods

### Case Selection

The Institutional Animal Care and Use Committee (IACUC) at Colorado State University (protocol number 1371) reviewed and approved the study. Written informed consent was obtained from the owners for the participation of their animals in this study. Young to middle aged horses initially intended to be used for performance, who were euthanized due to severe behavioral or training issues judged to be too dangerous to themselves or the rider, were included in the study. The owners' chief complaint and description of the concerning behaviors were recorded. All available records from previous diagnostic evaluations and applied treatments were reviewed and recorded. All horses had prior veterinary examinations and multiple treatment regimens that failed or were only effective for a short period. The region of concern from the owner's or treating veterinarian's perspective was typically localized to the axial skeleton, with a strong suspicion that the cervical region was the primary source of the pain and subsequent dangerous behavior.

### Spinal Examination

The spinal evaluation procedure included myofascial (i.e., soft tissue) palpation and vertebral mobilization and was performed by a dual-boarded equine surgeon and sports medicine and rehabilitation specialist certified in veterinary acupuncture and chiropractic (MS) (10). The myofascial examination was used to assess the behavioral responses to light touch, tone and texture of the superficial soft tissues, and development of the epaxial musculature (e.g., the semispinalis capitis, splenius, longissimus, iliocostalis, and middle gluteal muscles) (Supplementary Video 1). The skin was evaluated for the ability to glide freely over the underlying superficial fascia and muscles without any resistance or sensitivity. The soft tissues were palpated from superficial-to-deep, assessing signs of inflammation, such as heat and swelling, tissue texture, muscle tone, and areas of sensitivity within the different tissue layers (11). The quantity (e.g., affected tissues, area and depth) and the quality (e.g., severity) of any abnormal findings were recorded. The presence, location and severity of muscle asymmetry were also noted (12). The quantitative (e.g., range of motion) and qualitative (e.g., ease and fluidity) characteristics of both passive and active (i.e., baited carrot stretches) lateral bending of the cervical and thoracolumbar regions were recorded. Vertebral segment mobilization throughout the axial skeleton was assessed in lateral bending and flexion-extension (13). Compression of the tubera sacralia and bilateral ventral mobilization of the tubera coxae were performed to evaluate lumbosacral (LS) and sacroiliac (SI) joint motion and reactivity. Scapular motion was used to evaluate caudal cervical to cranial thoracic (C7-T4) mobility (cervical lateral bending and dorsal scapular motion) and pain (backing away, pulling the limb away, or rearing). The clinician induced dorsal scapular motion *via* passive elevation of the entire unweighted forelimb by holding the metacarpal region parallel to the ground while flexing the limb at the carpus. This was performed with concurrent ipsilateral passive lateral bending of the head and neck, to the extent the horse would allow, by an assistant. All parameters were graded as normal (no restriction), mild, moderate or severe restrictions within each spinal region.

### Behavioral Responses

Behavioral responses noted during the myofascial and spinal mobility examinations were graded (by MS) as normal, mild, moderate, severe, or too dangerous to evaluate (14). Normal behavior was characterized as no observable response to palpation or mobilization of any spinal region. Mild behavioral responses consisted of slight reactions to deep pressure, the presence of mild hypertonicity or stiffness (lack of normal range of motion of joints or flexibility of soft tissues), and a single spinal region affected. A moderate behavior score was assigned if the horse reacted to moderate pressure, the presence of moderate hypertonicity or stiffness, and if several spinal regions were affected. Moderate behavioral reactions included the horse holding the ears back behind vertical, attempts at biting or kicking, and moving away from the examiner. Severe behavioral reactions were noted in horses that were hyperreactive to any applied touch, would try to bite the handler, continuously move away from the examiner, and had a strong resistance to any induced spinal mobility within multiple vertebral regions. Horses categorized as too dangerous to evaluate displayed aggressive behaviors such as biting, kicking, striking and rearing, which prevented examiners from approaching or touching them.

### Gait Evaluation

The lameness examination (performed by MS) included walking and trotting in hand on hard ground in a straight line and in 15 meter circles in hand in both directions and was graded 0–5 per the AAEP lameness scale (15). The neurologic evaluations were performed by YNL (dual-boarded in critical care and internal medicine with clinical emphasis on equine neurologic diseases). The neurologic examination included a cranial nerve examination, walking in a straight line with the head held in neutral and elevated positions, walking in small circles to evaluate forelimb and hindlimb placement, backing the horse up for several strides, and evaluating the response to lateral tail traction while standing still and during walking. Additional neurologic challenges including walking the horse up and down a small incline and over a curb were included dependent on animal compliance and environmental circumstances. Ataxia was graded 0–5 based on the modified Mayhew scale (16). Dysmetria and weakness were graded as absent, mild, moderate, or severe.

### Diagnostic Imaging

All previous imaging studies performed at the Colorado State University Veterinary Teaching Hospital had been evaluated by board certified radiologists on clinical duty (MB, KS) and these records were evaluated and recorded. At the time of enrollment, an updated diagnostic imaging evaluation [radiographs, ultrasound, *ex-vivo* computed tomography (CT)] of the cervical region was completed. Cervical radiographic evaluation (Toshiba 1700) was performed by a blinded observer (MB), and included lateral-lateral radiographs of the occiput to the first thoracic (T1) vertebra. Oblique projections were also included as indicated to clarify abnormalities noted on the lateral cervical radiographs. Radiographic images were evaluated for enlargement of the articular process joints (APJ), (e.g., osteophyte and enthesophyte formation), and the size of the intervertebral foramina. Radiographic findings were graded as normal, mild, moderate or severe (17). Ultrasound examination (GE Healthcare, Logiq 90, 12 MHz linear probe, or the Toshiba Aplio i700, 10 MHz linear probe) of the cervical region was performed bilaterally from the occiput to T1 vertebrae (18). Ultrasonographic images were evaluated in real-time during image acquisition and consensus graded (MB, MS). The cervical APJ margins and joint capsule enthesis were graded as normal (no abnormalities), mild (small area of bone proliferation or irregular surface), moderate (moderate bone proliferation and irregular surface), and severely affected (substantial bone proliferation or fragmentation noted). The intervertebral foramina and presence or absence of joint effusion were also evaluated and graded as normal or abnormal. Joint capsule thickness was subjectively evaluated as normal or thickened. CT (Gemini Big Bore, Philips Healthcare, Andover, MA) was used for *ex vivo* evaluation of the cervicothoracic region (C1–T5). CT images were consensus graded by MS and a blinded, board-certified equine radiologist (KS) (19). The size of the APJs were graded as normal, mild, moderately or severely enlarged. The medial and lateral articular margins and the joint capsule were evaluated for the presence of osteophytes and enthesophyte, respectively. All regions were graded as normal, mild, moderate, or severely affected. The subchondral bone of the APJ was scored as either normal or irregular. The cervical intervertebral foramina were evaluated and graded as normal or narrowed, using the cranial and caudal foramina to compare size. The nerve roots were graded as normal or enlarged. The width of the intervertebral disc (IVD) space was graded as normal or narrowed, and the dorsal profile of the IVD was graded as normal (no protrusion), mild, moderate, or severely protruding.

### Gross Pathologic Examination

Horses were euthanized by intravenous administration of barbiturates (Pentobarbital 390 mg/ml, 60 ml) and immediately transported to the necropsy facility. The head and cervical region were collected en bloc *via* transection at the T5-T6 vertebral level and immediately transported for CT imaging. Following CT imaging, the soft tissues were removed from the cervicothoracic specimen, which was sectioned through the vertebral bodies within the frontal plane to allow en bloc removal and histologic processing of the spinal cord, nerve roots and dorsal root ganglia (DRG). Gross examination of the neural tissues included evaluation of the brain, spinal cord, venous plexus, and DRG (C1-T3) for alterations in color and presence of induration and/or nodularity. The DRG were considered abnormal when enlarged, firm and nodular. Attempts were made to routinely collect bilateral DRG from all cervical vertebral levels in all horses. When indicated based on clinical or lameness examinations, the ipsilateral brachial plexus (only the proximal brachial plexus, not the nerves contributing to the plexus) and the thoracolumbar, lumbosacral or sacropelvic regions were also collected and evaluated grossly for soft tissue, osseous and neural pathology. Gross examination of the osseous and articular structures was performed by a blinded observer (KH) who was aware of the general regions of concern within each horse, but who had no specific knowledge of the clinical or diagnostic imaging findings. The vertebral bodies were examined for the presence of spondylophytes and the IVD were examined grossly for signs of disc degeneration, which was subjectively graded as normal (no degeneration), mild, moderate and severe using a prior grading system (20). The joint capsules of the APJs were resected to allow complete visualization of the articular surfaces and joint margins for signs of articular cartilage damage, osteophyte and enthesophyte production, and modeling of the articular surfaces and joint margins (21). Articular changes were graded as normal, mild, moderate or severely affected using previously reported scoring methods (21). The amount of joint capsule thickening and presence of synovial fold hyperemia was also noted.

### Histopathology

The DRGs from the cervical, thoracolumbar, and lumbosacral regions were collected and processed for histologic evaluation. The DRG were bisected with one-half fixed in 10% neutral buffered formalin (NBF) and the other half fresh frozen (−80°F for future evaluation). Transverse and sagittal sections of dorsal nerves and ganglia were processed and embedded in paraffin. Sections were cut (5–6 μm) and attached to egg albumin-coated slides and then were stained using routine hematoxylin and eosin (H &E), Masson's trichrome, and Luxol's fast blue using the respective procedures established at the Colorado State University, Veterinary Diagnostic Laboratories (CSU-VDL). Sensory nerve rootlets and their corresponding DRG were histologically evaluated for cellularity, fibrosis, the formation of lymphocytic nodules or hyperplasia of satellite cells, and the presence of Nageotte nodules. A diagnosis of spinal neuritis (neuroinflammation of the nerve roots), ganglionitis or both was based on gross and histological evidence of pathological alterations. Evidence of ganglionitis was histologically established if there was: significant hypercellularity due to infiltration by increased numbers of lymphocytes (perivascular cuffs or endoneurial infiltrates), macrophages including clustering of hemosiderin-laden histiocytes, hyperplasia of satellite cells, fibrosis and drop-out of ganglionic neurons (22). Fibrosis was further confirmed through staining with Masson's trichrome. Drop-out of ganglionic neurons was evident as many neurons were almost totally obliterated by neuronphagia. DRG from non-affected regions (i.e., most commonly C1-C2) served as intra-horse histologic controls. DRG from three clinically (riding-age horses, euthanized for owner-related reasons, not showing any signs of allodynia or hyperalgesia or having a history of axial skeleton concerns) and histologically normal horses served as histologic references (unpublished data; TA and KH). Control ganglia from each corresponding horse or clinically normal (no pain on clinical examination) control horses had a total score of <400 nucleated cells (excluding neuronal and fibroblasts). Mildly affected ganglia had increased cellularity >400 <500 cell/high power microscopic field (HPMF); moderate ganglionitis >500 <600 cell/HPMF; and severe ganglionitis >600 cell/HPMF. Based on clinical examination findings, the proximal brachial plexus (the individual nerves were not evaluated separately) in select cases was evaluated. The brachial plexus was considered normal if the bundles of nerves were merged together and ensheathed with perineurium with a regular contour. There was no nodular infiltrate in the endoneurium or epineurium. As has been described in laboratory animals (23), signs of inflammation of the plexus included perineural fibrosis and multifocal perivascular cuffs comprising moderate numbers of lymphocytes, plasma cells and hemosiderin-laden macrophages.

### Clinical Case Summaries

An aggregate assessment was used to determine the primary spinal region of concern. The aggregate was formed using all available information which included the medical history, spinal examination and behavioral response, gait evaluation, diagnostic imaging results, and gross and histopathologic evaluations.

## Results

### Horses and Owner Complaints

Fourteen horses that developed severe performance limitations and dangerous behaviors under saddle that eventually resulted in euthanasia were included in this case series. The mean age was 9.4 ± 2.6 years and included eleven geldings and three mares. The breeds included eight warmbloods, four Thoroughbreds, one Quarter Horse, and one Andalusian. Eight of the horses were used for dressage, four for eventing, one for show hunting, and one for barrel racing. The owners' chief complaint and reports of dangerous behavior were recorded (Table 1). In 12 horses, the time from purchase to euthanasia was 2.5 ± 1.8 years. The other two horses were raised by their owner, and the time from initial complaint to euthanasia was less well-defined. Ten (71%) owners' primary complaint was of dangerous behavior. Professional riders rode the remaining four horses and the primary complaint was performance limitations; two riders complained of aggressive behavior, one falling down and one for bruxism and inability to back up.

**Table 1:** Presenting owner complaint and the interval of time from purchase to euthanasia.

**Horse**	**Age at euthanasia (years)**	**Complaint**	**Owner concerns**	**Interval (in years)**
1	8	Performance limitations	Bruxism undersaddle Shivers-like behavior, not able to back up Unable to advance in training	7^*^
2	9	Dangerous	Resistant to go forward Stumbling on the front limbs Rearing under saddle	1
3	7	Dangerous	Dangerous behavior under saddle	2
4	12	Dangerous	Violent, dangerous behavior under saddle	2.5
5	12	Dangerous	Resistance under saddle, difficult to collect Some rearing, progressed to falling down Suspected seizure activity	5^**^
6	9	Dangerous	Unable to lower head, not able to get into a dressage frame Violently throws head and panics while being ridden Nearly fell on the owner during a painful episode Bucked the owner off a twice	3
7	10	Dangerous	Bucking and explosive bolting under saddle	2
8	6	Dangerous	Resistance under saddle, won't move forward Bucking and rearing	NA
9	12	Performance limitations	Aggressively tossing head Falling down and collapsing which caused secondary trauma and multiple wounds	1.5
10	6	Dangerous	Intermittently unable to lower his head to the ground Leaping around, spooky and unrideable	2
11	14	Performance limitations	Hypersensitive Difficult to canter	1
12	11	Dangerous	Pinning ears, seems uncomfortable Progressed to dangerous behavior, unable to ride	1
13	10	Dangerous	Unpredictable and very nervous from the right side Unable to advance in training Trainer refused to ride	NA
14	6	Performance limitations	Reluctant to move forward especially at the canter Aggressive behavior	1.5

* *Owner initially complained of training difficulties as a yearling*.

** *Owned for 1.5 years prior to the initial presentation with a complaint of resistance to work and rearing. NA, Not available because these horses were raised from foals by the respective owners and underwent multiple periods of training and rest due to ongoing concerns*.

### Spinal Examination and Behavioral Responses

Eleven (79%) horses were extremely reactive to initial palpation and light touch of the skin and superficial fascia (Supplementary Table 1). Ten (71%) horses exhibited severe behavioral responses throughout the myofascial and mobilization examinations, which included kicking, biting, moving away, striking or rearing, which made physical examination difficult and dangerous (Supplementary Videos 2–4). One horse displayed severe avoidance and dangerous behavior (i.e., too dangerous to evaluate) that precluded any additional palpation or mobilization examination. The remaining three (21%) horses displayed moderate behavioral responses (e.g., bracing to palpation or subtle increased tension in the muscle, ears back, tension in the eyes). All horses which were palpated [(13); one horse was unable to be palpated due to the dangerous behavior) showed some degree of abnormal reactivity to palpation of the left and/or right brachiocephalicus muscle(s) (Supplementary Table 1). Five (38%) horses displayed moderate to severe signs of reactivity to palpation and mobilization of the withers and scapular regions. Ten (77%) horses had moderate to severe abnormalities localized to the thoracolumbar region, which included hypertonic epaxial muscles and resistance to mobilization in lateral bending or flexion and extension. Seven (54%) horses had moderate to severe adverse reactions to axial compression of the tubera sacralia, ventral mobilization of each tuber coxa, or stimulated pelvic flexion. Five (38%) horses had reactivity to palpation at the proximal attachment of the semitendinosus muscle (unilateral or bilateral, Supplementary Table 1) along the lateral aspect of the sacrum (S3–S5).

### Gait Evaluation

Thirteen (93%) horses displayed lameness in at least one fore or hind limb. No horse had greater than grade 3 lameness in any limb (subtle, but present, lameness noted in a straight line). Seven horses displayed bilateral forelimb lameness, three had unilateral forelimb lameness, three horses had a unilateral hind limb lameness, and four horses had both fore and hind limb lameness. A complete neurologic examination was completed in 13 of the 14 horses (one horse was not available for a neurologic examination before euthanasia). None of the horses displayed signs of pelvic limb ataxia. One horse displayed bilateral thoracic limb hypometria when walked, and when walked with a raised head this dysmetria worsened. No pelvic limb gait abnormalities were noted in this horse. One horse showed mild thoracic limb and moderate pelvic limb weakness, and one horse showed signs of mild hypermetria in both pelvic limbs.

### Diagnostic Imaging

Diagnostic imaging modalities used over the year(s) prior to enrollment into the study included radiography, ultrasonography (percutaneous and transrectal), and nuclear scintigraphy. These imaging modalities were utilized during lameness evaluations before acceptance into the study and results were available for ten horses (Supplementary Table 2). Prior diagnostic imaging included cervical (*N* = 8) and thoracolumbar radiographs (*N* = 5). Ultrasonographic examinations of the axial skeleton included cervical (*N* = 4), thoracolumbar (*N* = 5) and transrectal (*N* = 8) evaluations. Mild (*N* = 4) or moderate (*N* = 2) periarticular bone proliferation of the APJs, at various levels from T16-L6, was noted in all horses with thoracolumbar ultrasound examination. Transrectal ultrasound examination (24, 25) revealed two horses with periarticular bone proliferation of the right SI, and one horse had bilateral SI proliferation. Five horses had abnormalities noted at the LS junction which included narrowing of the LS disc space, fibrosis or mineralization of the LS disc, or L6 endplate remodeling. Due to poorly localized musculoskeletal pain or dysfunction, six horses underwent full body skeletal scintigraphy examination. On nuclear scintigraphy, one horse had mild, diffuse increased radiopharmaceutical uptake in the region of the C4-C5 APJ, and one had moderate, diffuse increased radiopharmaceutical uptake in the region of the C6-C7 APJ (26). Five horses had increased radiopharmaceutical uptake in the thoracolumbar spinous processes (*N* = 4) or the APJ (*N* = 3), and three horses had increased radiopharmaceutical uptake in the sacrum and/or ilium close to the right SI joint.

At the time when owners elected euthanasia, the horses were enrolled in the study. All horses had updated diagnostic imaging of the cervical spine, including radiographic, ultrasonographic, and *ex-vivo* CT imaging (Supplementary Table 2). On radiography, eight horses had mild (*N* = 5) or moderately (*N* = 3) enlarged cervical APJs and one horse had narrowed C6-C7 intervertebral disc space. Ultrasonographic evaluation revealed APJ periarticular bone proliferation; mild (*N* = 5), moderate (*N* = 5) and severe (*N* = 1) at multiple levels between C2-T1. CT examination of the cervical region revealed enlargement of the APJs (*N* = 6), periarticular proliferation (*N* = 12) of the APJ, and IVD protrusion at multiple levels between C2-T2: mild (*N* = 6), moderate (*N* = 6) and severe (*N* = 1). Lobular, hyperattenuating regions on CT examination were interpreted as epidural hemorrhage at C1-C2 (*N* = 1) and T4 (*N* = 2), and subarachnoid hemorrhage T4-T5 (*N* = 1).

### Gross Pathologic Examination

The initial horse enrolled into the study lacked a detailed gross examination of the cervical vertebral column. Within all subsequent enrolled horses (*N* = 13), moderate to severe osteophytes of the cervical APJ (*N* = 11) and moderate to severe intervertebral disc disease (*N* = 7) localized to the caudal cervical region (Supplementary Table 3) were evident. Dural hemorrhage at C7-T1was noted in two horses.

In horses that the thoracic vertebral column was grossly evaluated (*N* = 8), dural hemorrhage within the cranial thoracic region (*N* = 2), and moderate intervertebral disc degeneration (*N* = 3), were noted. Impingement of the spinous processes at T11–T17 was noted in two horses, with moderate APJ modeling at the same vertebral levels of the impingement. The lumbar vertebral column was evaluated in ten horses with intertransverse joint ankylosis noted at L4-L5 (*N* = 1) and L5-L6 (*N* = 4), and moderate to severe L4-L6 APJ modeling (*N* = 4). Narrowed intervertebral disc space and or disc protrusion was noted from L5-S1 (*N* = 4) with dural hemorrhage present at L4-L6 in one horse.

The sacrum and pelvis were evaluated grossly in twelve horses, with moderate to severe SI joint modeling (*N* = 7) and lumbar sacralization (*N* = 3) noted. Dural hemorrhage at the lumbosacral junction was seen in four horses.

### Histopathologic Examination

**Figure 1:**
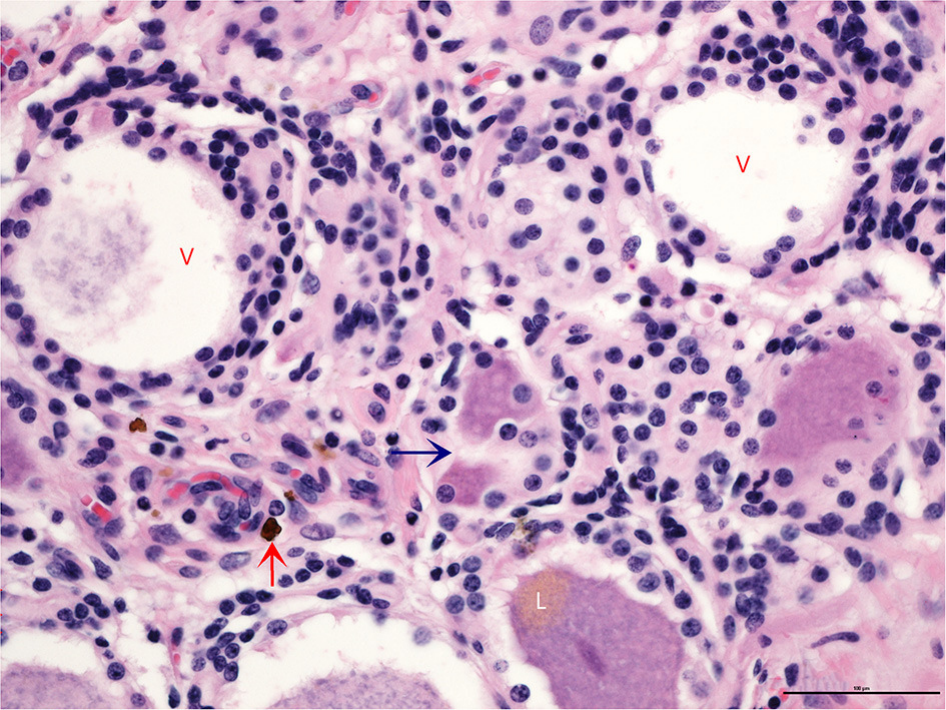
Transverse section of a dorsal root ganglion showing neuronal cell bodies and their corresponding axons. Partial to complete absence of cytoplasm is evident in the larger vacuolated neurons (V), which are surrounded by increased numbers of satellite cells that appear to encroach upon the periphery of perikarya engulfing degenerate soma along with fewer microglia (blue arrow). The lower neuron shows polar accumulation of brown pigment granules, lipofuscin (L), an incidental finding. Vessels are cuffed by small numbers of hemosiderin-laden macrophages (red arrow). Overall cellularity of the ganglionic stroma is significantly increased (*N* = 723) (H and E, 400x).

All horses in this case series (*N* = 14) had multiple levels of moderate to severe ganglioneuritis (Supplementary Table 4). In contrast, the DRG in control horses (*N* = 3) had normal cell counts and lacked evidence of any pathologic changes. In affected DRG, the ganglionic neurons (perikarya) had partial to total clearing of perinuclear cytoplasm (chromatolysis). Within the moderately and markedly inflamed ganglia, drop-out of neurons and replacement with fibrosis was evident. In the most affected neurons, nuclei were shrunken and hyperchromatic (pyknotic). Surrounding scalloped perikarya were several layers of hyperplastic satellite cells, which encroached upon degenerate or necrotic perikarya forming >3 Nageotte bodies per a 400x HPMF (Figure 1). Histologic evidence of brachial plexitis (i.e., inflammation of the brachial plexus; *N* = 4) and acute hemorrhage (epidural to subdural; *N* = 9) was noted. No other clinically significant findings were noted within the central nervous system during the post-mortem examination.

### Clinical Case Summaries

The aggregate assessment of the most commonly affected vertebral region included the cervicothoracic region (*N* = 7), lumbosacral region (*N* = 5), cervical (*N* = 1) and an aggregate assessment could not be made in one horse because the dangerous behavior disallowed palpation (Supplementary Table 5). Moderate to severe ganglionitis was noted within the aggregate region of interest (*N* = 12). Based on gross post-mortem examination, the lumbosacral region was the most severely affected region. Across all horses, the cervical and cervicothoracic regions were judged to be the most commonly affected sites (Supplementary Table 5). The thoracolumbar region was not considered the primary region of concern within any horse.

## Discussion

This case series suggests that the dangerous behavior noted in these horses was more likely due to the presence of neuropathic (i.e., structural) pain rather than due to “bad” behavior or poor training. We hypothesized that physically identifiable lesions would be found within the cervical region at post-mortem examination (i.e., gross and histopathologic evaluation), that would explain the observed adverse behaviors despite extensive, yet inconclusive, prior diagnostic imaging, and the lack of response to numerous applied treatments. In preliminary work, routine gross and histologic evaluation of the central nervous system in six horses (not included in this series) failed to reveal any clinically significant pathologic findings within the brain or serial sections of the spinal cord; therefore, we began to expand our search to the DRG, spinal nerve roots, and nerves. Ganglionitis alone or ganglioneuritis was diagnosed in all horses within this case series; however, the DRG lesions were not specifically localized to the cervical region. As the clinical study progressed, it became apparent, based on painful responses to myofascial and mobility examinations throughout the entire axial skeleton, that all spinal regions should be thoroughly evaluated at necropsy. In research models, it has been shown that the DRG may be affected several spinal segments from a site of structural pathology (27). Therefore, we expanded our search to include the entire axial spine in order to capture gross and histologic information that would have been overlooked if our investigation was limited only to the cervical region.

The DRGs contain the cell bodies of afferent sensory neurons and serve an important role in relaying peripheral sensory information to the central nervous system (28). DRG are located within, or close to, the intervertebral foramina, the size of which may be compromised by changes in posture or the presence of adjacent soft tissue or osseous proliferation (e.g., osteophytes or disc protrusion). This may then lead to DRG injury which could cause the neurons to become hyperexcitable, which in turn might result in spontaneous firing (28). Satellite glial cells (SGC) are found wrapped around the neuronal cell bodies in the DRG where they play a role in neuronal homeostasis. When there is nerve damage or inflammation, the SGC become activated, resulting in neuronal hyperexcitability and consequent pain (29). The vasculature of the DRG is permeable, unlike the blood-brain barrier present within the central nervous system (30). This lack of barrier allows local inflammatory mediators (i.e., secondary to IVDD or APJ OA) direct access to the DRG and subsequent activation of the SGC (29–31). These cellular and molecular responses at the level of the DRG, secondary to inflammation or nerve damage, facilitate chronic pain (32).

In humans, ganglionitis has been documented in chronic, neuropathic pain syndromes (32, 33). Similarly, ganglionitis has been reported in chronic, pathologic pain syndromes in horses associated with laminitis and idiopathic forelimb lameness (34–38). In this case series, all horses had moderate to severe ganglionitis identified at multiple vertebral levels which we theorize to be related to the observed dangerous behavior and apparent neuropathic pain. However, a thorough evaluation of the cellular and molecular markers from DRG acquired from a normal sports horse population would greatly improve our interpretation of the clinical relevance of these histopathologic findings.

The cervicothoracic region (C7-T4) was the most common region identified as the site of pain and dysfunction, followed by the lumbosacral junction (L6-S1) and then cervical region (C2-C7). The neuroanatomical localization of neuropathic pain to the cervicothoracic region (C7-T4) and brachial plexus was based on the presence of forelimb lameness and ipsilateral reactivity to dorsal scapular mobilization combined with ipsilateral bending of the neck. Interestingly, brachial plexus injuries are highly associated with the presence of neuropathic pain in humans and include inflammatory brachial plexopathies and plexitis due to idiopathic, traumatic (39), viral, bacterial and immune-mediated mechanisms (40). Similar inflammatory and immunologic mechanisms need to be explored in horses.

A common clinical finding in this case series (71%) was identifying subdural and epidural hemorrhage and/or hematomas, that were most commonly present at the cervicothoracic and lumbosacral junctions. In humans and dogs, epidural and subdural hematomas occur (41–44), and are considered surgical emergencies (45). In contrast to this case series, pain associated with spinal hematomas in humans and dogs is usually acute, intense, and generally associated with neurologic deficits caused by spinal cord compression (42, 46). In similar fashion to our report, spontaneous epidural hematomas in humans occur more commonly in the high mobility areas of the cervicothoracic and thoracolumbar spine (47). Epidural hematomas localized to the cervicothoracic region have been reported in the equine literature. However, the horses in that report all displayed ataxia attributed to spinal cord compression associated with the hematoma (48). In that series, Gold et al. reported hematomas to be chronic in nature, as fibrin and hemosiderin-laden macrophages were evident in those lesions. The etiology and pathogenesis of spinal hematomas in human medicine is difficult to define as many different classifications have been used such as; spontaneous, idiopathic, traumatic, coagulopathic, and other (42). Similar mechanisms that may affect vascular fragility within the vertebral canal need to be explored in horses.

Pain is defined as “an unpleasant sensory and emotional experience” (49) and is categorized as nociceptive, inflammatory or pathological (50). Nociceptive pain is protective and serves to limit contact with noxious stimuli through the withdrawal reflex. Inflammatory pain, often as a result of injury or surgical intervention, is also protective and commonly managed with the administration of NSAIDs. Pathological pain is not protective and can be divided into neuropathic pain (i.e., structural neural lesion) or dysfunctional pain (i.e., neuropathologic functional disorder) (50, 51). Pathologic pain, without an inflammatory component, is unlikely to respond to NSAIDs. Hyperalgesia, defined by the International Association for the Study of Pain (IASP), is “increased pain from a stimulus that normally provokes pain,” and allodynia is pain caused by stimulation that does not typically cause pain (52), both are frequently associated with neuropathic pain (53). Neuropathic pain in humans can be spontaneous, and does not need to be associated with ongoing tissue damage (54). This has been described in horses (37, 55) and may also be true in this series.

Given that most horses accept human touch, 71% of horses is in this report were judged to be allodynic. As has been proposed in human medicine (53), signs of allodynia and hyperalgesia may be useful indicators of the presence of neuropathic pain in horses (56). In addition to the importance of clinical examination findings, pain questionnaires in human medicine have also been shown to be helpful in the diagnosis of neuropathic pain (57). In equine practice, it is also critically important to listen to the rider or owner describe their horse's behavior to increase awareness of subtle pain behaviors (58, 59). In this series, the owner and trainer's initial complaint and historical account were very similar in nature. There was a common thread of recent purchase (i.e., within 3 years) with the intent for each horse to be used for athletic endeavors, but never able to achieve the level of intended use due to the development of dangerous behavior. There was also a failure to resolve dangerous training issues after multiple diagnostic and therapeutic attempts, which together should alert veterinarians to the possibility of severe underlying disease processes and the potential development of associated neuropathic pain. The authors believe this lack of response to routine therapies (e.g., NSAIDs, intra-articular corticosteroid treatments) is an important indicator that a horse may be experiencing neuropathic pain. In contrast, dangerous behavior that is readily modified with diagnostic analgesia is much more likely to be due to inflammatory pain (3).

Most diagnostic imaging modalities are limited to providing a pathoanatomic diagnosis; however, a collection of medical history and clinical examinations, in addition to diagnostic imaging findings, may help to identify underlying disease mechanisms that may contribute to the development of neuropathic pain. In the cervicothoracic region, vertebral endplate sclerosis and narrowing of the intervertebral disc as seen on radiographs, or protrusion of the dorsal intervertebral disc identified on CT support the diagnosis of chronic vertebral instability and IVDD (55). In humans, IVDD has been associated with the development of neuropathic pain (60). The radiographic diagnosis of impinged thoracolumbar spinous processes may be of little clinical significance in some horses (61, 62), but is likely an important radiographic finding in horses that have notable pain responses to digital palpation along the dorsal midline, epaxial muscle hypertonicity or atrophy, and severe avoidance behavioral responses to spinal mobilization (e.g., flexion-extension) of the affected spinal region. The ventral aspect of the lumbosacral region is frequently evaluated with transrectal ultrasonography; (63) however, varying degrees of irregular intertransverse or sacroiliac joint margins can be noted in horses that do not have obvious clinical signs of pain or dysfunction localized to this region. More recently, changes in intervertebral disc echogenicity at L5-L6 and the lumbosacral intervertebral levels have been associated with regional pain and poor performance (64). Similarly, horses in our study with reactivity localized to the lumbosacral region often had evidence of L6 sacralization, ankylosis of the lumbar intertransverse joints, or lumbar and sacroiliac osteoarthritis. These horses would frequently buck or kick during manual palpation of the lumbar epaxial muscles, consistently unlock both stifles during tubera sacralia compression, and have severe adverse reactions to ventral mobilization of the tubera coxae.

When presented with complicated cases such as these, an aggregate assessment, which incorporated the primary findings across all outcome parameters and owner complaints, proved useful in defining a specifically affected spinal region. In this case series, the histopathologic evaluation was considered the gold standard for a definitive diagnosis and localization of the neurologic lesions. As hypothesized, organic lesions of the nervous system were identified, however there was no single antemortem modality or examination finding that clearly indicated the exact site of the pathoanatomic lesions in this case series. While it is likely that there is a causal relationship between the clinical features and post mortem findings, this is difficult to determine without more control horses and grading of lesion severity.

## Limitations

A limitation of this study is the lack of complete data sets within each horse. As a case series, there was clinical variation in the available retrospective data available. Additionally, inadvertent oversight or the inability to collect gross and histologic tissue samples at all vertebral levels and the brachioplexus prevented complete analysis in some horses. We used three control horses to provide comparisons; however, the incorporation from additional unaffected (non-painful) age-matched sports horses would have helped to expand our understanding of the relationship between ganglionitis and pain-behavior.

## Conclusion

The purpose of this case series is to raise awareness and acknowledge that severe behavioral problems in horses may be due to lesions of the nervous system resulting in neuropathic pain. This case series highlights the need for a more in-depth understanding of pain behavior and its clinical presentation and progression in severely affected horses that do not respond to traditional therapies used to treat musculoskeletal pain or lameness. The client and trainer perspectives are critically important to recognizing pain behavior. The myofascial and spinal mobility examinations provided critical information to identify clinical signs that justified the horse's unwanted or dangerous behavior, and helped to localize the spinal regions of interest. When this localization has been established, advanced diagnostic imaging modalities may be instituted, focusing on the highly mobile cervicothoracic, thoracolumbar and lumbosacral regions.

The overall objective is to develop an early diagnosis and effective treatment of neuropathic pain syndromes in horses so that they may live full, productive, and pain-free lives.

## Data Availability Statement

The original contributions presented in the study are included in the article/Supplementary Material, further inquiries can be directed to the corresponding author.

## Ethics Statement

The animal study was reviewed and approved by Institutional Animal Care and Use Committee. Written informed consent was obtained from the owners for the participation of their animals in this study. Written informed consent was obtained from the individual(s) for the publication of any potentially identifiable images or data included in this article.

## Author Contributions

MS, TA, KH, YN-L, and CM contributed to study design and review of the manuscript. MS contributed to the acquisition of cases, review of records, myofascial and lameness examinations, review of diagnostic imaging, and manuscript preparation. KH and TA performed the post-mortem examinations (gross and histopathology). YN-L performed neurologic examinations. MB and KS provided diagnostic imaging analysis. All authors contributed to the article and approved the submitted version.

## Funding

This study was supported by the Leslie Malone Presidential Chair in Equine Sports Medicine.

## Conflict of Interest

The authors declare that the research was conducted in the absence of any commercial or financial relationships that could be construed as a potential conflict of interest.

## Publisher's Note

All claims expressed in this article are solely those of the authors and do not necessarily represent those of their affiliated organizations, or those of the publisher, the editors and the reviewers. Any product that may be evaluated in this article, or claim that may be made by its manufacturer, is not guaranteed or endorsed by the publisher.
