# Supplementary material for: Dangerous Behavior and Intractable Axial Skeletal Pain in Performance Horses: A Possible Role for Ganglioneuritis (14 Cases; 2014–2019)
Source: Front Vet Sci. 2021 Dec 10;8:734218. doi: 10.3389/fvets.2021.734218 (PMC8702524; doi:10.3389/fvets.2021.734218)
Supplement: Supplementary file 1 [file Table_1.docx]

**Table 1.** Grade of behavioral responses, response to light touch, and positive findings and response to palpation and joint mobilization within spinal regions. BL = Bilateral, LB = Lateral bending, FE = Flexion-extension, C = Cervical vertebrae, T = Thoracic vertebrae, L = Lumbar vertebrae, TL = Thoracolumbar junction (T18-L1), LS = Lumbosacral joint (L6-S1), TC = Tubera coxae, TS = Tubera sacralia, NSF = No significant findings, NE = Not examined for safety reasons.

|  |  |  |  | **Spinal Regions** |  |
| --- | --- | --- | --- | --- | --- |
| **Horse** | **Behavior** | **Touch** | **Cervical** | **Thoracolumbar** | **Lumbosacral and Sacropelvic** |
| 1 | Severe | Severe | Moderate pain BL brachiocephalicus m.  Mild stiffness BL LB | Moderate hypertonicity BL epaxial m.  Severe pain FE T10-L6 | NSF |
| 2 | Severe | Severe | Moderate pain BL brachiocephalicus m.  Mild stiffness left LB  Moderate stiffness right LB | Moderate stiffness right T16-T17 | Moderate focal pain right S3-S5 |
| 3 | Severe | Severe | Moderate pain left brachiocephalicus m.  Moderate stiffness right LB | Moderate stiffness right L3-L6  Severe pain right L3-L6 | Moderate focal pain BL S3-S5  Moderate pain right SI joint |
| 4 | Severe | Severe | Moderate pain BL brachiocephalicus m.  Moderate stiffness BL C4-C6 | NSF | NSF |
| 5 | Severe | No | Moderate pain BL brachiocephalicus m.  Moderate pain right poll region  Severe pain left scapular elevation | Severe pain left T8-T12  Severe hypertonicity left T10-L6  Moderate pain BL L1-L6 | Moderate focal pain BL S3-S5 |
| 6 | Incapacitated | Incapacitated | NE | NE | NE |
| 7 | Moderate | No | Moderate pain BL brachiocephalicus m. | Moderate pain right T4-T12  Moderate stiffness right T8-T12 | Moderate reaction TS compression |
| 8 | Moderate | Severe | Mild pain BL brachiocephalicus m. Moderate pain left splenius m. | Mild pain left T8-T12 | Mild reaction LS flexion  Mild focal pain BL S3-S5 |
| 9 | Severe | Severe | Severe pain BL brachiocephalicus m.  Mild stiffness BL LB | Moderate hypertonicity BL T10-L6  Severe reaction LB T12-T18 | Moderate focal pain BL S3-S5  Moderate pain TC mobilization |
| 10 | Severe | Severe | Moderate pain BL brachiocephalicus m.  Severe reaction right scapular elevation | Severe reaction LB T8-T12  Severe reaction extension T8-T16 | Moderate reaction TC mobilization |
| 11 | Severe | Severe | Severe pain poll region  Moderate pain BL brachiocephalicus m.  Moderate stiffness BL LB | Moderate hypertonicity BL T10-L6  Moderate reaction BL T8-T12  Moderate stiffness T10-L6  Severe reaction extension TL | Moderate reaction LS extension |
| 12 | Severe | Severe | Moderate pain BL brachiocephalicus m.  Moderate stiffness BL LB  Moderate reaction left scapular elevation | Severe pain BL T8-T18  Severe reaction LB T8-T18  Severe reaction FE T8-T18 | Severe pain LS flexion |
| 13 | Moderate | No | Moderate pain BL brachiocephalicus m.  Moderate stiffness right LB | NSF | NSF |
| 14 | Severe | Severe | Moderate pain BL brachiocephalicus m.  Moderate stiffness BL LB | Moderate reaction T8-L6  Severe reaction right LB L3-L6 | Moderate pain TS compression |

**Table 2.** Pathologic findings localized to anatomical structures within spinal regions based on diagnostic imaging modalities. The positive findings of the listed modalities are color coded: nuclear scintigraphy (purple), radiography (blue), ultrasonography (black), and CT imaging (red). AP = Articular process joint, R = Right side, L = Left side, C = Cervical vertebrae, T = Thoracic vertebrae, L = Lumbar vertebrae, S = Sacral vertebrae, IVD = Intervertebral disc, IVF = Intervertebral foramen, SI = Sacroiliac, SP = Spinous process, NE = Not Examined, NSF = No significant findings, Uptake = increased radiopharmaceutical uptake

|  |  | **Spinal Regions** |  |
| --- | --- | --- | --- |
| **Horse** | **Cervical** | **Thoracolumbar** | **Lumbosacral and Sacropelvic** |
| 1 | NSF  Mild enlarged AP C6-C7  Mild peri-articular bone proliferation AP C2-C4, C6-C7  Mild periarticular bone proliferation AP C2-T1  Mild non-articular irregular bone AP C2-C3  Moderate protrusion IVD C4-C5, C6-T1  Subchondral bone sclerosis (increased bone density) cranial AP C6-C7 | Moderate uptake SP caudal T16-T18  Moderate uptake AP caudal T16-T18  Moderate impinged SP T18-L2  Mild periarticular bone proliferation AP T16-L1 | Moderate uptake right SI joint  Moderate periarticular bone proliferation right SI joint  Mild periarticular bone proliferation left SI joint  Moderate fibrosis IVD L6-S1  Mild endplate remodeling L6-S1 |
| 2 | Mild uptake AP C4-C5  Mildly enlarged AP C6-C7  Mild non-articular irregular bone AP C2-C3  Narrowed IVD C6-C7  Mild lysis caudal endplate C6  Mild periarticular bone proliferation AP C3-C7  Mildly enlarged AP C3-C5, C6-C7  Mild to marked periarticular bone proliferation AP C3-C7  Mild non-articular irregular bone AP C2-C3  Mild irregular subchondral bone AP C2-C5  Moderate protrusion IVD C2-T1  Ventral narrowing and mineralization IVD C5-C7 | Mild uptake AP T17-T18 | NSF |
| 3 | Moderately enlarged AP C5-C7  Mild periarticular bone proliferation AP C4-T1  Mild enthesopathy AP C4-C6  Mild effusion AP C5-C7  Mild periarticular bone proliferation AP C4-C7  Mild enthesopathy AP C2-C5  Mild thickened joint capsule AP C5-C6  Mild protrusion IVD C6-T1 | NE | NE |
| 4 | Mild periarticular bone proliferation AP C2-C7  Mild periarticular bone proliferation AP C3-C4, C6-C7  Moderate enthesopathy AP C2-C3  Moderate periarticular bone proliferation AP C3-C6  Moderate enthesopathy AP C2-C4  Moderate subchondral bone irregularity AP C4-C6  Narrowed IVD C2-T1  Moderate protrusion IVD C4-C6  Narrowed IVF C2-C3  Enlarged nerve root C2-C3 | NE | NE |
| 5 | NSF  NA  NA | Mild sclerosis L3-L4  Moderate periarticular bone proliferation right L5-L6  Osseous irregularity IVF right L6 | NSF |
| 6 | NSF | Mild increased uptake SP T16-T17 | Mild diffuse uptake right SI joint |
| 7 | Moderate diffuse uptake AP C6-C7  Mild enlarged AP C6-C7  Mild periarticular bone proliferation AP C6-C7  Mild enthesopathy AP C4-C5  Moderate periarticular bone proliferation AP C3-C5  Mild joint capsule enthesopathy AP C2-C3  Thickened joint capsule AP C4-C5  Mild kyphosis C6-C7  Mild protrusion IVD C5-T1 | Mild uptake SP T13-T18  Mild uptake AP L1-L2  SP impingement T13-T18  Mild periarticular bone proliferation AP L1-L2  Mild kyphosis T1-T4  Osteochondral fragment AP T2-T3  Epidural hemorrhage T4 | Moderate diffuse uptake right SI joint  Mild periarticular bone proliferation right SI joint |
| 8 | Mild enlarged AP C6-C7  Moderate periarticular bone proliferation AP C6-C7  Severe periarticular bone proliferation AP C6-C7  Moderate enthesopathy AP C6-C7  Moderate effusion AP C6-C7  Mild effusion AP C5-C6  Thickened joint capsule AP C5-C7  Mildly enlarged AP C6-C7  Mild subchondral defect AP C2-C4, C5-C6, C7-T1  Mild periarticular bone proliferation AP C2-C3, C4-C6, C7-T1  Mild protrusion IVD C4-C5, C6-C7  Thickened joint capsule AP C3-C4  Transposition of ventral tubercle C6  Mildly narrowed IVF C4-C5 | Mild SP impingement T17-T18  Epidural hemorrhage T4 | NE |
| 9 | Moderately enlarged AP C4-T1  Moderate periarticular bone proliferation AP C6-C7  Moderate periarticular bone proliferation AP C5-C6  Mild effusion AP C6-C7  Moderate thickened joint capsule AP C6-C7  Mild periarticular bone proliferation AP C7-T1  Mildly irregular subchondral bone AP C2-C4  Mildly thickened joint capsule AP C3-C4  Moderately thickened joint capsule AP C6-C7  Mild enthesopathy AP C4-C5  Severe protrusion IVD C6-C7  Mild protrusion IVD C4-C5  Slightly enlarged nerve roots C6-C7 | Mild periarticular bone proliferation AP T18-L1  Moderate periarticular bone proliferation AP L1-L2  Mild periarticular bone proliferation AP T1-T2 | Mild periarticular bone proliferation BL SI joint  Moderate mineralization IVD L6- S1  Mild bone proliferation IVF L6 |
| 10 | NSF  Moderate periarticular bone proliferation AP C6-C7  Moderately enlarged AP C5-C6  Mild periarticular bone proliferation AP C3-C5, C6-C7  Mildly irregular subchondral bone AP C2-C6, C7-T1  Mild protrusion IVD C4-T1  Mild kyphosis and spondylosis C5-C6 | Mild periarticular bone proliferation AP T1-T2  Mildly narrowed IVD T1-T4  Moderate protrusion IVD T1-T2  Subarachnoid hemorrhage T4-T5 | NE |
| 11 | Mild enlarged AP C2-C3, C5-C7  Mild periarticular bone proliferation AP C2-C3, C5-C6  Moderate periarticular bone proliferation AP C2-C4  Mild periarticular bone proliferation AP C5-C7  Mild enthesopathy AP C2-C4  Mild effusion AP C6-C7  Mild periarticular bone proliferation AP C3-C4, C5-C6  Moderate protrusion IVD C3-C7  Epidural hemorrhage C1-C2  Moderate joint capsule enthesopathy AP C2-C3 | Mild periarticular bone proliferation AP L3-L4  Mild periarticular bone proliferation AP T1-T2 | Sacralization of L6 |
| 12 | NSF  NSF  Mild periarticular bone proliferation AP C2-C3, C4-C5  Moderate periarticular bone proliferation AP C7-T1  Mild effusion AP C7-T1  Mildly enlarged AP C3-C5  Mild periarticular bone proliferation AP C3-C7  Mildly irregular subchondral bone AP C2-C4  Mild protrusion IVD C3-C7 | Mild uptake SP mid to caudal T  Mild SP impingement T17-L2  Mild periarticular bone proliferation AP T18-L3 | NSF  Narrowed IVD L6-S1  Sacralization vertebra L6-S1 |
| 13 | Moderate enlarged AP C2-C3  Mild enlarged AP C5-T1  Mild periarticular bone proliferation AP C6-C7  Mild bone proliferation caudal occiput  Mild periarticular bone proliferation AP C2-C5, C7-T1  Mild enthesopathy AP C3-C4  Moderate thickened joint capsule AP C5-C7  Moderately enlarged AP C2-C5  Severely enlarged AP C5-C7  Moderate periarticular proliferation AP C2-C7  Severe irregular subchondral bone AP C4-C7  Moderate protrusion IVD C5-C7  Mildly narrowed IVF C2-C3, C4-C7 | NE | NE |
| 14 | NSF  NSF  Moderate periarticular bone proliferation AP C6-C7  Mild periarticular bone proliferation AP C4-C6, C7-T1  Mild enthesopathy AP C6-C7  Mild effusion AP C6-T1  Mildly enlarged AP C5-T1  Mild periarticular bone proliferation AP C2-C7  Mildly irregular subchondral bone AP C2-C3, C5-C6  Mild protrusion IVD C3-C4, C7-T1  Moderate protrusion IVD C4-C5  Mild kyphosis C6-C7  Mildly narrowed IVF C6-T1 | NSF | NSF  Severe narrowing IVD L6-S1  Sacralization of L6  Moderately narrowed IVF S1 nerve roots |

**Table 3.** Gross pathology findings within spinal regions. IVDD = Intervertebral disc degeneration, IT = Intertransverse, IVF = Intervertebral foramen, SP = Spinous process, AP = Articular process joint, BL = Bilateral, C = Cervical vertebrae, T = Thoracic vertebrae, L = Lumbar vertebrae, S = Sacral vertebrae, SI = Sacroiliac, NSF = No significant findings, NE = Not examined.

|  |  | **Spinal Region** |  |
| --- | --- | --- | --- |
| **Horse** | **Cervical** | **Thoracolumbar** | **Lumbosacral and Sacropelvic** |
| 1 | NE | Ankylosis L5-L6 | Narrowed vertebral canal L6-S1  Dorsal protrusion IVD L6-S1  Dural hemorrhage L6-S1 |
| 2 | Severe periarticular bone proliferation AP C7-T1  Severe IVDD C4-T1  Dorsal protrusion IVD C6-T1  Dural hemorrhage C7-T1 | Moderate spondylophyte T14-T16  Ankylosis L4-L5  Widened IVD L5-L6 | Narrowed IVD L6-S1  Severe periarticular bone proliferation BL SI joint |
| 3 | Moderate periarticular bone proliferation AP C2-C4 | Moderate SP impingement T16-T17  Moderate periarticular bone proliferation AP T16-T17  Moderate SP impingement L5-L6 | Moderate periarticular bone proliferation L6-S1 |
| 4 | Mild IVDD C3-C5  Moderate IVDD C6-C7  Severe IVDD C7-T1  Hemorrhage C7-T1 | NE | NE |
| 5 | Moderate periarticular bone proliferation AP C6-T1  Thickened joint capsule AP C3-T1  Severe IVDD C7-T1 | Moderate IVDD T3-T4 | Severe periarticular bone proliferation BL SI joint |
| 6 | Moderate periarticular bone proliferation AP C2-T5  Severe periarticular bone proliferation AP C7-T1 | Impinged SP T11-T17 | Osteochondroma right ilium |
| 7 | Severe IVDD C6-T1  Thickened joint capsule right AP C6-C7  Moderate IVDD C5-C6  Moderate periarticular bone proliferation AP C6-T1 | Dural hemorrhage T1-T2  Moderate IVDD T1-T2  Moderate periarticular bone proliferation IT joint L4-L5  Severe periarticular bone proliferation IT joint and IVF occlusion L5-L6  Ankylosis L5-L6 | Severe periarticular bone proliferation BL SI joint  Hemorrhage LS |
| 8 | Moderate periarticular bone proliferation AP C3-C5, C6-T1  Thickened joint capsule AP C3-C5, C6-T1  Malformation C3-C4 (pseudoarthrosis)  Hemorrhage C2-C3 | NE | NE |
| 9 | Moderate IVDD C2-C4  Severe IVDD C7-T1  Moderate periarticular bone proliferation AP C3-C6 | Moderate IVDD T3-T4  Ankylosis IT joint L5-L6 | Moderate periarticular bone proliferation and IVF occlusion L6-S1  Moderate periarticular bone proliferation right SI joint |
| 10 | NSF | Dural hemorrhage T4-T9  Spondylosis L3-L4  Narrowed IVD L5-L6  Ankylosis L5-L6 | Moderate periarticular bone proliferation and IVF occlusion L6-S1  Stress fracture left L6  Severe periarticular bone proliferation left SI joint  Dural hemorrhage L6-S1 and cauda equina |
| 11 | Hemorrhage right poll  Dural hemorrhage C1-C4  Moderate periarticular bone proliferation AP C2-C4  Severe IVDD C4-T1  Thickened joint capsule AP C6-T1 | Moderate periarticular bone proliferation  right IT joint L5-L6  Severe IVDD L5-L6  Severe protrusion IVD L5-L6 | Sacralization of L6  Severe periarticular bone proliferation BL SI joints  Dural hemorrhage L6-S1 and cauda equina |
| 12 | Moderate periarticular bone proliferation left AP C7-T1 | Moderate periarticular bone proliferation Right AP L5-L6  Epidural hemorrhage T1-T3 | Moderate periarticular bone proliferation BL SI joints  Sacralization of L6 |
| 13 | Moderate IVDD C3-C6  Moderate periarticular bone proliferation BL AP C2-C7  Severe periarticular bone proliferation right AP C5-C6 | Dural hemorrhage L4-S1 | NSF |
| 14 | Moderate periarticular bone proliferation AP C6-C7 | Mild periarticular bone proliferation IT joint L4-L5  Moderate periarticular bone proliferation IT joint L5-L6 | Sacralization of L6 |

**Table 4.** Histopathologic findings across spinal regions. NE = Not examined, C = Cervical vertebrae, T = Thoracic vertebrae, LS = Lumbosacral joint (L6-S1), S = Sacral vertebrae.

| **Horse** | **Cervical** | **Thoracolumbar** | **Lumbosacral and Sacropelvic** |
| --- | --- | --- | --- |
| 1 | Moderate ganglionitis C4-C6 | Severe ganglionitis T9-T18 | Moderate ganglionitis LS  Epidural hemorrhage LS |
| 2 | Moderate ganglionitis C4-T1  Epidural hemorrhage C7-T1 | Epidural hemorrhage T1-T2 | NE |
| 3 | Severe ganglionitis C2-T1 | Moderate ganglionitis T1-T4, T17-T18  Severe ganglionitis T11-12 | NE |
| 4 | Moderate ganglionitis C1-C6  Severe ganglionitis C6-C7  Epidural hemorrhage C7-T1 | Severe ganglionitis T1-T2 | NE |
| 5 | Severe ganglionitis C6-T1  Left brachial plexitis | Severe ganglionitis T2-T3 | NE |
| 6 | Severe ganglionitis C1-C2, C7-T1 | NE | NE |
| 7 | Moderate ganglionitis C3-C6  Severe ganglionitis C7-T1  Left brachial plexitis | Severe ganglionitis T1-T3  Epidural hemorrhage T5 | Severe ganglionitis LS  Perineural hemorrhage LS |
| 8 | Moderate ganglionitis C1- T1 | Severe ganglionitis T2-T3  Subdural hemorrhage T2-T3 | NE |
| 9 | Moderate ganglionitis C3-C5, C6-T1  Epidural hematoma C7-T1 | Moderate ganglionitis T1-T2 | NE |
| 10 | Moderate ganglionitis C3-C5,  Severe ganglionitis C7-T1 | Severe ganglionitis Left T1-T5  Epidural hemorrhage T3-T5 | Subdural hemorrhage LS |
| 11 | Moderate ganglionitis C1-C3, C5-C7  Severe ganglionitis C7-T1  Epidural hematoma C1-C4 | NE | Moderate ganglionitis LS  Hemorrhage sacral nerve roots |
| 12 | Severe ganglionitis C4-T1 | Severe ganglionitis T1-T5  Moderate ganglionitis Right L4-L5 | NE |
| 13 | Moderate ganglionitis C4-C5, C6-C7  Bilateral brachial plexitis | NE | NE |
| 14 | Severe ganglionitis C6-C7  Moderate ganglionitis C3-C4, C7-T1  Right brachial plexitis | Severe ganglionitis T1-T2 | Severe ganglionitis S3  Epidural hemorrhage S2-S3 |

**Table 5.** Compiled clinical case summaries. The most severe findings localized to an affected spinal region are listed for the spinal examination, diagnostic imaging and pathology examinations with a final aggregate assessment across columns of the primary spinal region judged to be causing the dangerous pain behavior. IVD = Intervertebral disc, IVDD = Intervertebral disc degeneration, NE = Not examined, NSF = No significant findings, BL = Bilateral, SI = Sacroiliac.

| **Horse** | **Spinal Exam** | **Imaging** | **Gross Pathology** | **Histopathology** | **Aggregate** |
| --- | --- | --- | --- | --- | --- |
| 1 | Thoracolumbar  Lumbosacral | Cervical  Cervicothoracic  Thoracolumbar  Lumbosacral  Sacroiliac joint | Lumbosacral | Thoracic  Lumbosacral | **Lumbosacral:**  Narrowed vertebral canal and IVD protrusion L6-S1, dural and epidural hemorrhage L6-S1 |
| 2 | Cervicothoracic | Cervical  Cervicothoracic | Cervical  Cervicothoracic  Thoracolumbar  Lumbosacral  Sacroiliac joint | Cervicothoracic | **Cervicothoracic:**  Severe IVDD C4-T1 with dorsal protrusion C6-T1, dural and epidural hemorrhage C7-T1 |
| 3 | Cervical  Lumbosacral | Cervical | Cervical  Thoracolumbar  Lumbosacral | Cervical  Cervicothoracic  Thoracic | **Lumbosacral:**  Moderate periarticular bone proliferation L6-S1 |
| 4 | Cervical | Cervical | Cervicothoracic | Cervical  Cervicothoracic | **Cervicothoracic:**  Severe IVDD C7-T1, severe ganglionitis C6-C7, epidural hemorrhage C7-T1 |
| 5 | Cervicothoracic  Thoracolumbar | Lumbar | Cervicothoracic  Sacroiliac joint | Cervical Cervicothoracic  Brachial plexus | **Cervicothoracic:**  Severe IVDD C7-T1, severe ganglionitis C6-T3, Brachial plexitis |
| 6 | NE | NSF | Cervical  Cervicothoracic  Thoracolumbar | Cervical  Cervicothoracic | NE |
| 7 | Cervicothoracic  Sacropelvic | Cervical  Sacroiliac joint | Cervicothoracic  Lumbar  Lumbosacral  Sacroiliac joint | Cervicothoracic  Brachial plexus  Thoracic  Lumbosacral | **Cervicothoracic**:  Severe IVDD C6-T1, severe ganglionitis C7-T1, dural hemorrhage T1-T2, brachial plexitis |
| 8 | Cervical  Cervicothoracic | Cervical | Cervical | Cervicothoracic | **Cervicothoracic:**  Severe ganglionitis T2-T3, subdural hemorrhage T2-T3 |
| 9 | Cervical  Thoracolumbar  Sacropelvic | Cervical  Lumbosacral  Sacroiliac joint | Cervical  Cervicothoracic  Lumbar  Lumbosacral  Sacroiliac joint | Cervical  Cervicothoracic | **Cervicothoracic:**  Severe IVDD C7-T1,  epidural hematoma C7-T1 |
| 10 | Cervicothoracic  Thoracolumbar  Sacropelvic | Cervical  Cervicothoracic | Thoracic  Lumbar  Lumbosacral  Sacroiliac joint | Cervicothoracic  Thoracic  Lumbosacral | **Cervicothoracic:**  Severe ganglionitis C7-T5, epidural hemorrhage T3-T5 |
| 11 | Cervical  Lumbosacral | Cervical  Lumbosacral | Cervical  Cervicothoracic  Lumbar  Lumbosacral  Sacroiliac joint | Cervical  Cervicothoracic  Sacral | **Lumbosacral:**  Sacralization of L6, severe periarticular bone proliferation BL SI joint, dural hemorrhage L6-S1, hemorrhage sacral nerve roots |
| 12 | Cervicothoracic  Thoracolumbar  Lumbosacral | Cervicothoracic  Lumbosacral | Cervicothoracic  Lumbar  Lumbosacral  Sacroiliac joint | Cervical  Cervicothoracic | **Lumbosacral**:  Sacralization of L6 |
| 13 | Cervical | Cervical | Cervical  Lumbosacral | Cervical  Brachial plexus | **Cervical:**  Severe periarticular bone proliferation C5-C6 |
| 14 | Lumbosacral | Cervical  Lumbosacral | Cervical  Lumbar  Lumbosacral | Cervical  Cervicothoracic  Brachial plexus  Sacral | **Lumbosacral:**  Sacralization of L6, severe ganglionitis S3, epidural hemorrhage S2-S3 |
| **Totals** | Cervical = 6  Cervicothoracic = 6  Thoracolumbar = 5  Lumbosacral = 5  Sacropelvic = 2 | Cervical = 11  Cervicothoracic = 4  Thoracolumbar = 1  Lumbosacral = 5  Sacropelvic = 3 | Cervical = 8  Cervicothoracic = 8  Thoracolumbar = 3  Lumbar = 6  Lumbosacral = 10  Sacropelvic = 7 | Cervical = 9  Cervicothoracic = 12  Thoracic= 4  Lumbosacral = 3  Sacropelvic = 2 | Cervical = 1  Cervicothoracic = 7  Lumbosacral = 5 |
